# Experimental Study of Halloysite Nanofluids in Pool Boiling Heat Transfer

**DOI:** 10.3390/molecules27030729

**Published:** 2022-01-23

**Authors:** Thong Le Ba, Ahmed Baqer, Mohammed Saad Kamel, Gyula Gróf, Vincent Otieno Odhiambo, Somchai Wongwises, Lezsovits Ferenc, Imre Miklós Szilágyi

**Affiliations:** 1 Department of Inorganic and Analytical Chemistry, Faculty of Chemical Technology and Biotechnology, Budapest University of Technology and Economics, Muegyetem Rakpart 3, H-1111 Budapest, Hungary; eng.ahmedbaqer@gmail.com (A.B.); vodhiambo@edu.bme.hu (V.O.O.); szilagyi.imre.miklos@vbk.bme.hu (I.M.S.); 2 Department of Mechanical Techniques, Al-Nasiriya Technical Institute, Southern Technical University, Al-Nasiriya 64001, Thi-Qar, Iraq; kamel86@stu.edu.iq; 3 Department of Energy Engineering, Faculty of Mechanical Engineering, Budapest University of Technology and Economics, Műegyetem rkp.3, H-1111 Budapest, Hungary; lezsovits@energia.bme.hu; 4 Center for Energy Research, Konkoly-Thege Miklós út 29–33, H-1121 Budapest, Hungary; grof.gyula@ek-cer.hu; 5 Department of Mechanical Engineering, Faculty of Engineering, King Mongkut’s University of Technology Thonburi, Bangmod, Bangkok 10140, Thailand; somchai.won@kmutt.ac.th; 6 National Science and Technology Development Agency (NSTDA), Pathum Thani 12120, Thailand

**Keywords:** nanofluids, boiling, halloysite, heat transfer

## Abstract

Halloysite nanotube (HNT) which is cheap, natural, and easily accessible 1D clay, can be used in many applications, particularly heat transfer enhancement. The aim of this research is to study experimentally the pool boiling heat transfer (PBHT) performance of novel halloysite nanofluids at atmospheric pressure condition from typical horizontal heater. The nanofluids are prepared from halloysite nanotubes (HNTs) nanomaterials-based deionized water (DI water) with the presence of sodium hydroxide (NaOH) solution to control pH = 12 to obtain stable nanofluid. The nanofluids were prepared with dilute volume concentrations of 0.01–0.5 vol%. The performance of PBHT is studied via pool boiling curve and pool boiling heat transfer coefficient (PBHTC) from the typical heater which is the copper horizontal tube with a thickness of 1 mm and a diameter of 22 mm. The temperatures of the heated tube surface are measured to obtain the PBHTC. The results show an improvement of PBHTC for halloysite nanofluids compared to the base fluid. At 0.05 vol% concentration, HNT nanofluid has the best enhancement of 5.8% at moderate heat flux (HF). This indicates that HNT is a potential material in heat transfer applications.

## 1. Introduction

With the quick increase of the global population and the development of industries, the demands for resources have grown significantly [1]. Energy is recognized as the most important issue people are facing now and in the future. Therefore, more efficacious and beneficial energy usage and administration are needed [2]. Another reason comes from the ever-increasing rates of heat emission at both macro- (e.g., car engines) and micro-level (e.g., chips in computer and mobile phones). At the present time, the cooling by using two phase pool boiling processes is an essential matter in the industrial sector due to the urgent need for miniaturization in one hand and increasing the performance improvement of such heat exchange systems in another hand. The latent heat of vaporization during this process could play an important role in removing large amount of heat from high heat flux devices. Heat transfer (HT) requires an appropriate cooling system for effective heat dissipation with a competent working fluid. The conventional HT fluids currently used, like ethylene glycol, water, air, and oil, have very low thermophysical properties and low thermal conductivity (TC) compared to solids. The investigation of the new fluids with higher energy efficiency for HT is necessary to solve the HT challenges in heat exchange systems. Moreover, all methods for improvement of HT such as turbulence generation, area expansion are limited by the low TC of the fluids. Consequently, it makes sense that the improvement of TC behavior of fluids will be studied. Utilizing nanoparticles to make suspension has been an idea for many years. These fluids are called nanofluids with higher thermal properties than conventional fluids [3]. Many studies on their HT have been performed [4,5,6,7,8].

PBHT process is an important method in heat exchange systems and the industry in general. Based on the up-to-date research and the published works, PBHT process in the nucleate boiling regime is a complicated phenomenon. PBHT from tubes has been under consideration from HT professionals as it is used in many industrial fields such as reactor cooling, evaporators, boiler tubes, etc., [9]. The use of nanofluids in PBHT has shown conflicting results in the previous studies regarding PBHTC. It has been widely reported in the literature that PBHT performance is affected by numerous parameters and conditions, i.e., base fluid, nanoparticle type, nanoparticle shape and size, concentration volume, thermo-physical properties of base fluid, nanomaterial morphology, nanofluid stability, heating surface characteristics, and many more.

In 1986, experimental research with Al_2_O_3_ nanofluids (0.1–0.5 wt%) was performed by Yang et al. [10] to study the behavior of PBHT. It was presented that PBHT of nanofluids was improved in comparison to the base fluid. Das et al. [11] did an experimental study on using the alumina nanoparticles to see their performance and effect on the heat transfer coefficient (HTC) and pool boiling curve with the aid of using a narrow heated pipe. The result revealed that the system for boiling bubbles differs from small to standard industrial tubes because the bubbles are sliding from the ground part to the upper region. Research on PBHT of multi-walled carbon nanotube (MWCNT) nanofluids was made by Sarafraz et al. [12] for modified heating surfaces. In their study, by investigating some properties of diamond-shaped micro-finned surfaces, the results stated that the PBHTC debased on smooth surfaces, and with modified surfaces, it was enhanced to 77% for 0.3 wt%. Nevertheless, a continual layer was formed, and it caused a considerable decrease in HTC.

The PBHTC of ZnO nanofluid with the base fluid of ethylene glycol and DI water in a cylindrical vessel under barometric pressure was studied by He et al. [13]. It was found that the increase in HF significantly increases the HTC of the nanofluid. Xing et al. investigated the effects of surface modification with covalent and non-covalent functionalization groups on the PBHT of MWCNT nanofluids (0.1–1 wt%). It was seen that the PBHTC enhancements were 34.2% and 53.4% for MWCNT-COOH and MWCNT-OH nanofluids, respectively. This happened because the surface modification reduced the deposition of MWCNT. The impact of depositing Ag nanomaterials in re-entrant inclined mini-channels for PBHT was investigated experimentally by Akbari et al. [14]. It was found that with the rising concentration of the nanofluids to get a nanocoated polished surface, the PBHTC was higher. Moreover, the PBHT was improved by inclination and reentrancy compared with polished copper. The PBHTC of DI water and Al_2_O_3_ based nanofluids with low concentrations (0.0007 and 0.007 vol%) were studied by Manetti et al. [15]. The results showed an enhancement of HTC up to 15% for rough surfaces and 75% for the smooth surfaces compared to DI.

The PBHT performance of Fe_3_O_4_/DI water nanofluid was investigated by Salimpour et al. [16] under atmospheric pressure on a flat copper surface in order to investigate the impacts of concentration and type of the nanofluids, sedimentation thickness, roughness of the surface, and HF on the surface roughness after boiling experiments. It was seen that after the boiling test on the rough surface, the nanofluids’ PBHT decreased at low heat fluxes (HFs) and increased at high HFs, while on the smooth surface, the PBHT increased at low HFs and did not change at high HFs. The influence of surface roughness and nanoparticle deposition on surface wettability, contact angle, and PBHTC is determined. The higher concentration of Fe_2_O_3_ nanofluids increases the surface roughness. They acquired the greatest HTC for the smooth surface with the deposition of nanoparticles at low weight concentrations.

The PBHT of two kinds of distilled water-based nanofluids (CuO nanofluids and TiO_2_ nanofluids) on a flat heater plate was studied by Karimzadehkhouei et al. [17]. It was shown that the TiO_2_ nanofluids had the greatest improvement (around 15%) at the lowest concentration (0.001 wt%), while the enhancement of the CuO nanofluids was 35% for 0.2 wt% concentration. ZnO-EG nanofluids were used by Kole and Dey [18] for the experimental investigation of PBHT performance. The PBHT performance of ZnO nanofluids was studied for different concentrations at atmospheric pressure. The PBHTC of the nanofluid at 1.6 vol% was enhanced to 22% in comparison to that value of the base fluid. Kamel et al. [19] investigated the improvement of pool boiling heat transfer coefficient PBHTC using Al_2_O_3_ and CeO_2_ hybrid nanofluids based deionized water at atmospheric condition. Their heater geometry was horizontal typical copper tube which was fixed inside pool of working fluids that were tested to this purpose. They found that the PBHTC of the hybrid nanofluids was improved about 1.37 times higher than that of deionized water. However, they concluded that using hybrid nanofluids with the range of dilute concentrations utilized in this study could improve the heat transfer comparing with mono fluids (Alumina nanofluid or ceria nanofluid) with the initial surface roughness used in their study.

HNT is cheap, natural, easily accessible 1D clay having the chemical formula Al_2_Si_2_O_5_(OH)_4_.nH_2_O with *n* = 0–2 [20]. Halloysite can be widely used in catalysts, food and personal products, pharmaceuticals, cosmetics, also, to be utilized as molecular sieves in many processes like separation of liquids and gaseous mixtures, purification of water in refining industries [21,22,23,24]. It was shown that nanofibers or nanotubes have the greatest TC. Because of this, a lot of research on the TC enhancement of carbon nanotubes in nanofluids was performed [25]. Halloysite is a potential material in nanofluid applications. However, the study on HNT nanofluids was presented in few papers [26,27]. The nanofluids were prepared at different volume concentrations (from 0.5 to 1.5 vol%). These nanofluids were stabilized for more than one day using surfactant and medium of pH = 12. Their thermal properties were measured at different temperatures (from 20 to 80 °C). At 5 vol% concentration, HNT nanofluids have a TC of 8%. The results presented that HNT nanofluids have upstanding properties as heat transfer fluid with moderate viscosity and high thermal conductivity. Research on the application of these nanofluid is needed. However, there are no papers indicating the research of halloysite in the field of PBHT.

From the previous reported works, the main aim of this paper is to investigate the pool boiling heat transfer PBHT performance using the novel halloysite nanotubes with deionized water as a conventional fluid through testing, analyzing, and examining the behavior of the pool boiling process from typical horizontal copper heater with 22 mm outside diameter. However, the current study intends to explore the performance of PBHT and the very first-time utilization of novel prepared HNT nanofluids with different range of volume concentrations (0.01, 0.05, 0.1, and 0.5 vol%). The heat flux HF used in this investigation is from 15 to 117 kW/m^2^ in order to examine the impact of this novel nanofluid on the nucleate pool boiling regime which considered as a complex region during this phenomenon.

## 2. Materials and Methods

### 2.1. Materials

HNT mineral from Turplu/ Balıkesir, Turkey was used in the previous study [27]. 1 M NaOH was bought from Sigma-Aldrich (Saint Louis, MO, USA), while DI water was provided by the Department of Inorganic and Analytical Chemistry laboratory, Budapest University of Technology and Economics (Budapest, Hungary).

In the preparation of nanofluid, the two-step method was used in this experimental work to make the HNT nanofluids with different volume concentrations (0.01, 0.05, 0.1, and 0.5 vol%) and by adding the HNTs to base fluid with pH = 12 medium [26], then homogenizing the nanofluids with ultrasonication process.

### 2.2. Pool Boiling Experimental Test

**Figure 1:**
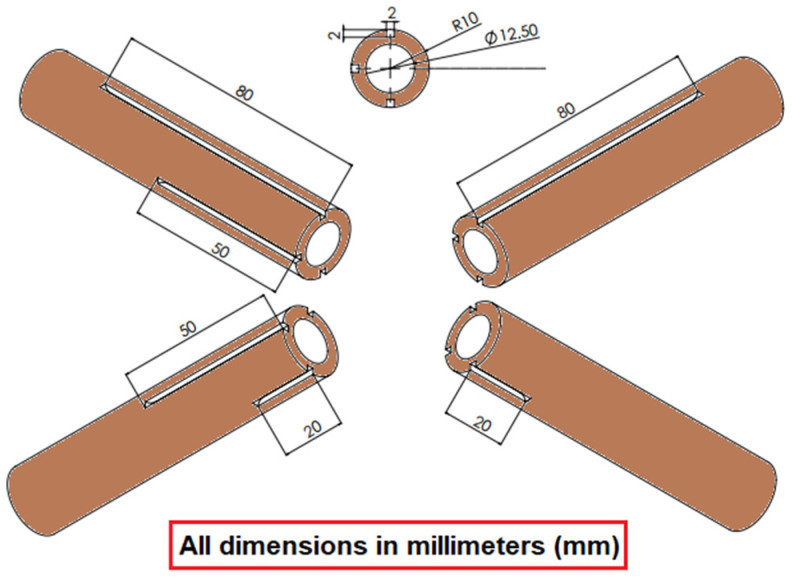
The isometric view of the copper sleeve used in this study.

**Figure 2:**
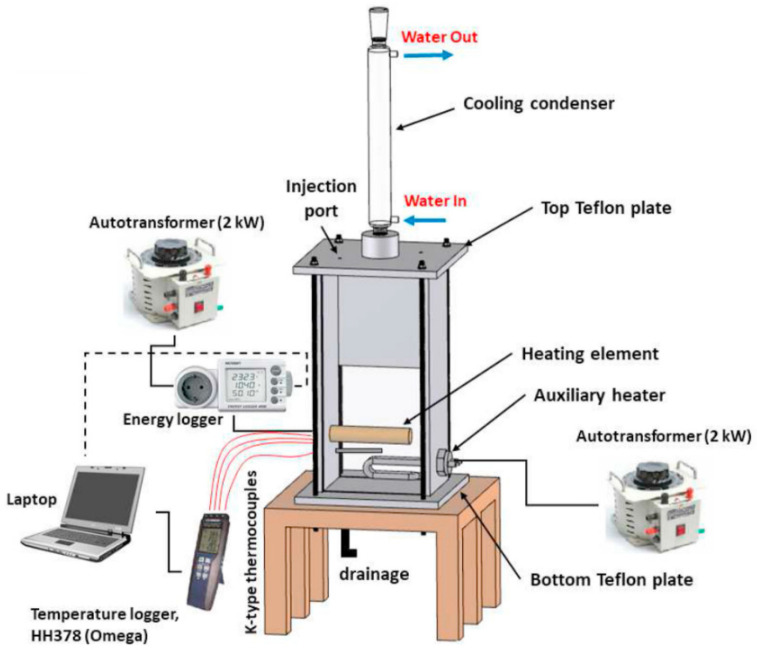
Schematic picture of the pool boiling device [19], reprinted from Elsevier with the Creative Commons CC-BY license.

The sleeve is constructed of a solid copper shaft with three holes designed at various radial angles and positions across the axial width as shown in Figure 1. The bulk and surface temperatures around the heating medium are measured using four K-Type thermocouples that have been previously tuned [28]. Another heating element was placed inside the chamber to provide more heating sources. Figure 2 shows the complete apparatus parts. The dimensions of this cylindrical tube are 22 mm external diameter and 1 mm thickness. The tube is filled with copper sleeves manufactured in the laboratory. The chamber pool is made of stainless steel metal with the following dimensions (length = 155 mm, width = 120 mm, and height = 310 mm). The type of the used reflux condenser (Allihn type NS29/32) has a 400 mm long jacket.

### 2.3. HNTs Properties

**Figure 3:**
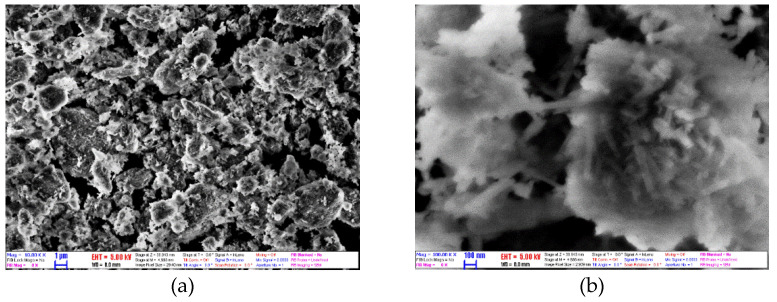
(**a**) 10,000× (**b**) 100,000× magnification SEM image of HNTs.

**Figure 4:**
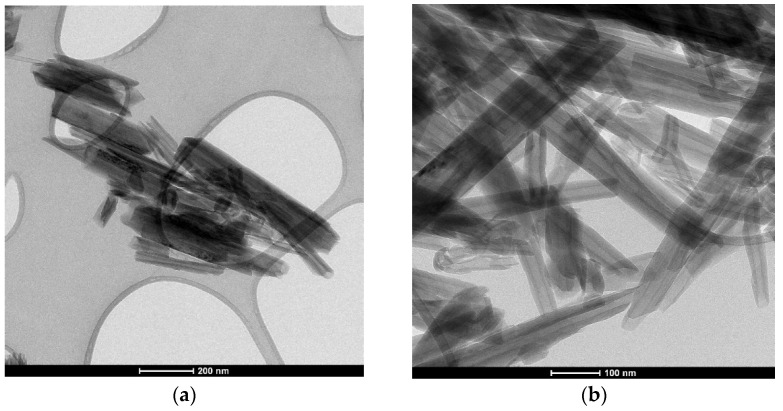
TEM images of HNTs (**a**) scale bar, 200 nm (**b**) scale bar, 100nm.

The HNT dry powders are examined by using a LEO 1440 XB scanning electron microscope (SEM) (LEOGmbH, Oberkochen, Germany) at 5 kV with a secondary electron detector at a high vacuum mode and Philips CM20 transmission electron microscope (TEM) (Philips, Eindhoven, Netherlands) at 200 kV to know the morphology and structure of the powder. In Figure 3, two SEM pictures are presented at different magnification powers. In Figure 3a with the 10 K(x) zoom, the morphology appeared to be bulky conglomerates. This showed that HNTs tend to stick together. Figure 3b with 100 K(x) clearly shows and confirms the tubular structure that has been suggested in the literature. Furthermore, the dimensions of the HNTs are as follows: the length is in the range of 100–300 nm, while the diameter is ca. 30–50 nm. Figure 4 shows two TEM images of HNTs. Based on this figure, it confirms the tubular morphology of the used halloysite.

**Figure 5:**
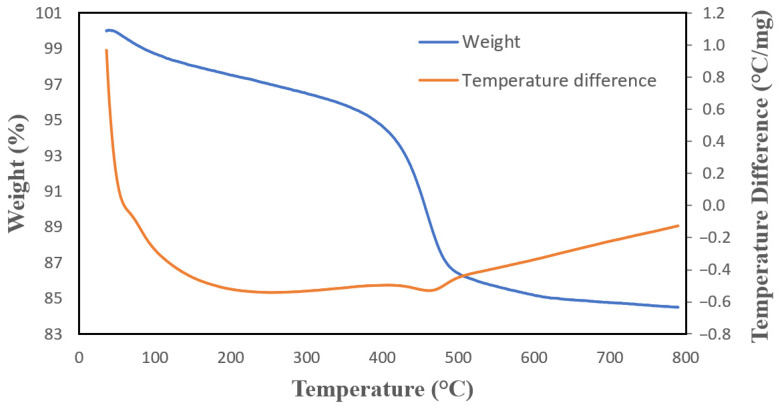
TG/DTA curve of halloysite in air.

The HNT sample is investigated via thermogravimetry/differential thermal analysis (TG/DTA) instrument 2960 SDT (TA Instruments Inc., New Castle, DE, USA) simultaneously between 20–900 °C at a heating rate of 10 °C/min. Figure 5 shows the thermal analysis of halloysite in the air. As it is suggested in the literature the imperial formula of halloysite is Al_2_Si_2_O_5_(OH)_4_.nH_2_O. Explaining the TG/DTA data, it can be seen that the sample lost 4.45% of its weight at 350 °C and mass loss continues until 400 °C which is due to the dehydration and the liberation of inner hydroxyl groups. At 410 °C to 565.5 °C, aluminum oxide (Al_2_O_3_) and silicon dioxide (SiO_2_) are formed in an endothermic reaction.

**Figure 6:**
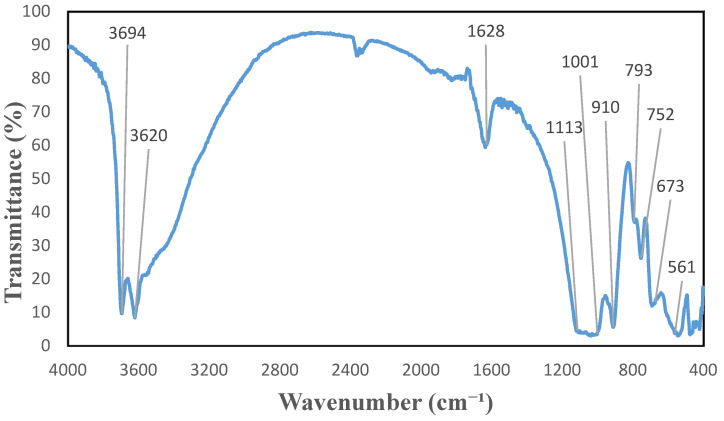
FTIR spectrum of HNT dry powders.

KBr pellets are used to characterize HNT powders using Excalibur FTS 3000 Bio-Rad Fourier-transform infrared spectroscopy (FTIR) equipment (Bio-Rad, Digilab, UK) in the region of 400–4000 cm^−1^ with a resolution of 1 cm^−1^ in transmittance. In Figure 6, FTIR spectrum of halloysite can be seen. All peaks below 600 cm^−1^ are said to be due to stretching, bending, and a combination of Si-O-Si. Al-O-Si bonds vibrations can be seen with a peak at 561 cm^−1^. The peak at 752 cm^−1^ is said to be due to the vibration of Al-O-Si [29]. At 910 cm^−1^ the vibration of inner hydroxyl groups is presented. Strong signaled stretching of Si-O-Si bonds can be observed at 1001 cm^−1^. It also can be seen that inner hydroxyl groups are presented at 1628 cm^−1^ as well. The peak 3620 cm^−1^ is also due to the inner hydroxyl (O-H) groups [29].

### 2.4. Digital Optical Microscopy (DOM)

**Figure 7:**
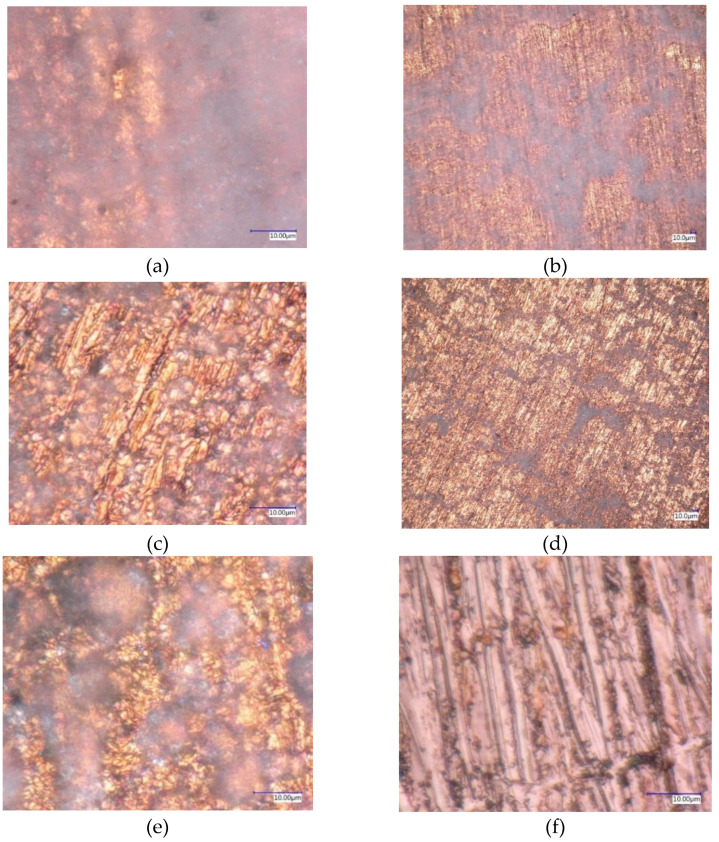
Deposition of the nanoparticles (**a**) 5000× for 0.5 vol%, (**b**) 500× for 0.5 vol%, (**c**) 5000× for 0.05 vol%, (**d**) 500× for 0.01 vol%, (**e**) 5000× for 0.1 vol%, and (**f**) 5000× Clean sample (without deposition).

In literature, there are some types of surface analysis such as SEM, TEM, and etc., [16]. In this study, the measurements of the digital optical microscopy were carried out with the Keyence VHX-5000 digital optical microscope (Keyence, Itasca, IL, USA). In Figure 7, there are the DOM measurement pictures for the four-volume concentrations (0.01, 0.05, 0.1, and 0.5 vol%) with 500× and 5000× magnification. From the pictures, it is clearly seen what the difference is between the clean sample (free of deposition) and the samples with the nano-powder deposited on the external layer of the heating tube; also, it can be noticed that the deposition ratio varies from one sample to another depending on the volume concentration. This deposition caused a negative effect on the HT process as it made a nanoporous layer which led to the deactivation of the nucleation site of the bubble formation.

## 3. Results and Discussion

### 3.1. Validation of Pool Boiling Chamber

**Figure 8:**
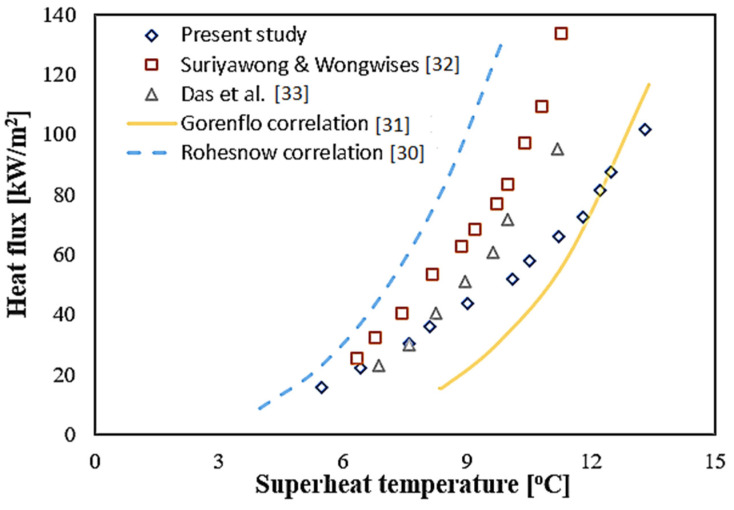
Validation of pool boiling curves of DI with Suriyawong and Wongwises [32], Das et al. [33], Gorenflo et al. [31] and Rohesnow et al. [30].

In order to confirm the reliability of the boiling chamber, DI water is used for investigation on PBHT. Then, the results of the pool boiling cure for DI water are compared with the previous studies [30,31,32,33], as shown in Figure 8. From the results, it can be seen that the experimental results in the present study can be accepted.

### 3.2. Pool Boiling of Halloysite Nanofluids

**Figure 9:**
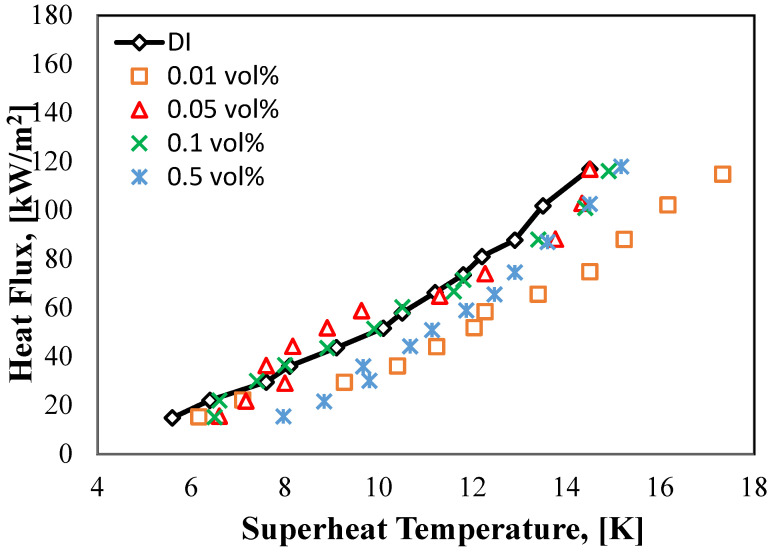
Pool boiling curves of DI water and HNT nanofluids at different concentrations.

Figure 9 shows the relationship between HF against the superheat temperature (∆Tsup = Tw − Tsat). This kind is defined as the pool boiling curve for the base fluid, and HNT nanofluids. Because of the power restriction of the cartridge heater utilized in this study, the maximum applied HF employed for all experiments does not surpass 118 kW/m^2^, which indicates that all experiments are performed below the critical HF value. As shown in the figure below, as the applied HF was raised, the superheat temperature has risen up with a fairly minor difference in temperature for both of the halloysite nanofluid and DI, and that is because of the nucleate pool boiling mechanism, which the vaporization heat represented. Moreover, it can be noticed that at the 0.05 vol% concentration the curve moves to the left side of the graph as compared to DI water at HF values ranging from 36.3 to 58.8 kW/m^2^ with superheat temperatures values 7.6 to 9.6 K. Moreover, there was a very slight enhancement with 0.1 vol% concentration curve, as it can be seen about the minor left shifting in its curve at HF values from 30 to 60.3 kW/m^2^ with superheat temperatures values 7.4 to 10.5 K and after these values as both of the HF and the superheat temperatures further increase, the curves start to move to the right side of the graph, and that means a deterioration in the HT process. However, increasing the concentration of the nanofluid significantly changes the boiling curve toward the right. Exceptionally, with high HF values, the shift is remarkable. Moreover, as the process was running forward, HF and superheat temperature kept rising, and at the same time, the pool’s boiling performance worsened. This might be linked to the nanoflakes accumulating on the surface of heating tube under the boiling phase. The result is that an insulation thermal layer preventing the HT between the heating tube and the working fluids is created. This explanation was used by several researchers [18,32,34].

**Figure 10:**
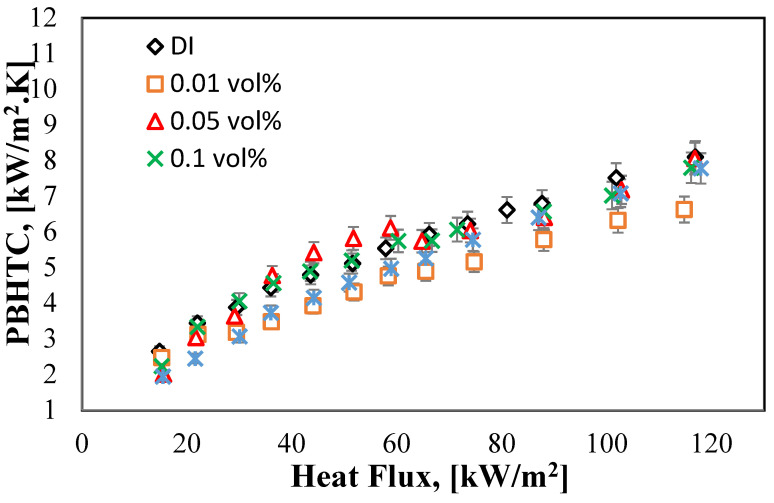
PBHTC against the applied HFs for DI water and halloysite nanofluid of four-volume concentrations.

Figure 10 shows the proportional relationship between the HF, q [kW/m^2^], and PBHTC, [kW/m^2^K]. From the graph, it can be told that there is an improvement in the PBHTC for the volume concentrations of (0.1% and 0.05%). The enhancement of 0.1 vol% curve as compared to DI water started at 30 [kW/m^2^] HF and 4.05 [kW/m^2^K] to reach the maximum enhancement PBHTC of 5.74 [kW/m^2^K] at 60.3 [kW/m^2^] of the HF. While the 0.05 vol% curve has the best HT process among all the other concentrations, as it increased by moving to the left side of the graph when the HF value reached 36.27 [kW/m^2^] with 4.77 [kW/m^2^K] of PBHTC and continued rising until it hits the highest value of the PBHTC at 6.10 [kW/m^2^K] with 58.8 [kW/m^2^] HF. However, after reaching the peak, the curves for both the abovementioned concentrations started to deteriorate as the boiling process moved forward with time. The reason behind this increment is related to the good TC of the halloysite nanofluid as well as the small volume concentrations of the nanofluids (0.1% and 0.05%) that helped in increasing the HT.

**Figure 11:**
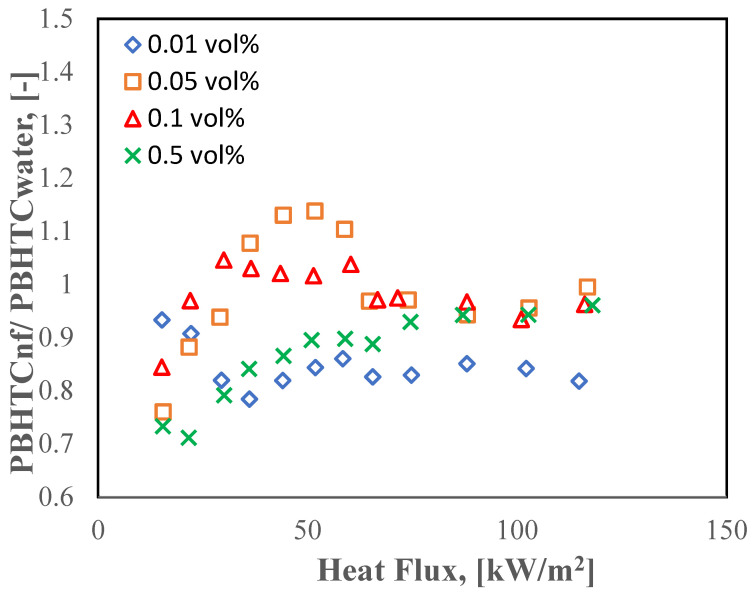
PBHTC ratio of HNT nanofluid and DI water at different concentrations and applied HFs.

Figure 11 shows the plotted graph between the applied HF and the ratio of PBHTC of halloysite nanofluid with respect to DI water (PBHTC_nf_/PBHTC_di-water_) for four volume concentrations. It can be clearly seen from the graph that the ratio of 0.05 vol% HNF has the best enhancement of 5.8% at moderate HF (51.74 KW/m^2^). Moreover, there is another improved ratio (about 5.7%) for the 0.1% dilute concentration at low HF (30 KW/m^2^). Furthermore, the behavior of the PBHTC of the nanofluid at moderate and low heat was discovered to be superior to that of water, particularly when it comes to volume concentrations (0.05% and 0.01 vol%), and that is because the bulk effect prevails in the free convection area.

The maximum reduction ratio of the PBHTC of HNT nanofluid with respect to DI water (PBHTC_nf_/PBHTC_di-water_) is for 0.5% HNFs with a ratio of (2.4%). Another notable piece of information from the figure is that at HF > 60 kW/m^2^ for most concentrations, the PBHT ratio degraded and the reason for this deterioration is the deposition of the solid material (dry powder nanoparticle) on the heating element surface, where the cavities are filled with the nanotubes of the HNTs and deactivate the nucleation sites, as it can be seen from Figure 7 at the digital optical microscopy (DOM) pictures. Here we can see the deposition of the nano-powder of 0.5 vol% concentration has covered the whole bottom part of the heating tube and due to the formation of bubbles in this part of the pipe, it slides to the sides of the heating tube and eventually leaving to the upper part of the chamber as explained in details by [33]. Another explanation was made by Kim et al. [34] on the deposition of the suspended solid material on the external surface of the heater inside the chamber, by showing that this deposition formed an insulation layer (nano-porous) and hindered the HT process, which resulted in making the heating tube hydrophilic. Because of the cohesion of the nano-powder to the heating surface, it has been postulated in this research that the accumulation of nanotubes alters the surface properties.

## 4. Conclusions

In this work, the performance of PBHT is experimentally examined by using four different dilute concentrations (0.01%, 0.05%, 0.1%, and 0.5%) of HNT based DI nanofluid with the aid of a horizontal copper tube as a heating element. Before the pool boiling test, the dry nanopowder was first characterized by various characterization techniques i.e., FTIR, Raman spectroscopy, SEM, and TG/DTA to investigate the morphology and structure of the HNT powders. The copper heating tube surface roughness is tested after each volume concentration measurement, and the arithmetic means roughness parameters (Ra) are 0.638 μm, 0.6 μm, 0.538 μm, and 0.193 μm and at the same time. The heater tube surface is also tested by using the DOM with different magnifications. The pictures showed the deposition of the nanopowder on the tube.

PBHT test is performed under atmospheric conditions. Results showed an improvement for halloysite nanofluid with these dilute volume concentrations (i.e., 0.05 and 0.1 vol%), which might lower superheat temperatures for different HFs, raised the PBHTC causing the boiling curve to move to the left. For 0.05 and 0.1 vol% at moderate HF, the maximum PBHTC ratio for HNT nanofluids is about 5.8% and 5.7% respectively, it is thought that this rise is linked to bulk effects represented by the improvement of the TC of the nanofluid.

## Data Availability

Not applicable.

## Sample Availability

Samples of the compounds are not available from the authors.

## Nomenclature

HNT	Halloysite nanotube
DI water	Deionized water
HT	Heat transfer
HTC	Heat transfer coefficient
MWCNT	Multi-walled carbon nanotube
PBHT	Pool boiling heat transfer
PBHTC	Pool boiling heat transfer coefficient
TC	Thermal conductivity
HF	Heat flux
